# A General View of the Results Obtained by Subcutaneous Surgery

**Published:** 1855-08

**Authors:** 


A General View of the Results obtained by Subcutaneous Surgery. By M. Guerin.—In a paper submitted to the Académie des Sciences, M. Guerin declares that all his subsequent experience is only confirmatory of the statements made by him in his celebrated memoir in 1839. In a general review of the subject, he here re states in a summary manner, the principles upon which subcutaneous surgery is based, and the practical applications it is susceptible of.

1. *Tissues when divided under the skin undergo immediate organization.*—In the first memoir, he showed that if the absence of contact of the air, and hermetical closure of the orifice of the incision were secured, immediate organization, with an absence of local and general reaction, resulted. He now adds that there are certain incidental conditions which, if not provided for, may interrupt this process, and cause failure even when the main condition of exclusion of the air has been observed. In this point of view the behaviour of the *fluids* of the economy is of importance. Some of these, as *arterial* blood, are organizable, and while a moderate quantity effused between the lips of the wound acts as an important element of their junction, even large quantities thrown out under the skin are absorbed with great rapidity. Other fluids, as *venous* blood, are *inorganizable*, and while portions of that effused may be resorbed, the remainder continues to offer a mechanical obstacle to immediate organization. Then, there are excreted fluids, such as bile, urine, and pus, which are *antipathetic* to immediate organization. Pus, confined under the skin may either undergo chemical change, or retain its normal characters. In the former case, the smallest portion will immediately excite reaction, and in the other gives rise, by inocculation, to the secondary formation of small cold abscesses.

The cicatrical tissue resulting from the healing of ordinary wounds bears no resemblance to any of the normal tissues, and constitutes a functional interruption in the organs in which it is deposited. In the immediate organization which takes place after subcutaneous wounds, all divided tissues are susceptible of producing their like at the cut surfaces, muscle, muscle, nerve, nerve, and so on, each endowed with its proper power. When, however, from the effusion of too much blood by surrounding vessels, the divided surfaces are kept too far separated, and the proper blastema is not exuded, functional interruption ensues here also, the extremities of the cut tissues becoming atrophied and losing their specific organization. This is the case with muscle, tendon, nerve, etc., but in none so obviously as with arteries, which are sometimes converted into fibrous cords for the whole length of the limb, contrasting with the integrity of their calibre when the contiguity of the divided surfaces has been maintained. The lameness which has often resulted from section of the tendo-Achillis, and the loss of motion of the fingers almost always produced by a division of the flexor tendons, are dependent upon the production of heteromorphous tissue consequent upon the non-observance of some of the conditions necessary for immediate organization. So, too, in the operation of strabismus, the functions of the muscles, when divided in an exposed state, are more or less impaired.

2. *Surgical Applications.*—Although tendons had already been divided by operations somewhat analogous to those of M. Guerin this was an emperical procedure only, having at most the object in view of limiting the amount of resulting inflammation. M. Guerin claims to have raised these subcutaneous operations into a method, based upon principles deduced from the teachings of experiment, and clinical and pathological observation. The *object* of this method is not to merely limit the amount of suppurative inflammation, but to absolutely prevent it, and to secure immediate organization by homogeneous tissue. The *means* of effecting this are comprised in four principal rules :—1. Make the wound in the skin at the greatest possible distance from that of the tissue to be divided, so that the track joining the two may be sinuous; 2. Circumscribe the section to the tissues to be divided, isolating them from surrounding parts by tension, contraction, etc.; 3. Prevent the effusion of inorganic or antipathetic fluids into the wound and expel all such, as well as air, from its track after the operation; 4. Close the lips of the external wound securely by adhesive plaster. The results, when the conditions of the operation are observed, are so satisfactory, that M. Guerin declares that, in many more than 2,000 cases, he has never met with a suppurating subcutaneous wound. In conclusion, he gives a summary of the various applications he has made of this method.

(A.) *Subcutaneous Sections* :—1. *Of the skin and cellular substance.*—In five instances, M. Guerin has detached bridles of cellular tissue, which by displacing the skin, or causing its adhesion have produced grevious deformities of the face or neck. 2. *Tendons.*—These operations are now so common as to call for no remark, further than indicating the superiority of the results attained compared with those of ordinary tenotomy. 3. *Aponeuroses.*—Beside the section of these as an orthopædic procedure, M. Guerin has resorted to it with success in *débridement* in inflammatory tumefaction. One of the most original applications of subcutaneous tenotomy was its employment in old and congenital dislocations, and the success that attended this practice has led to its adoption in aid of the reduction of recent fractures and dislocations. 4. *Muscles.*—The largest as well as the smallest muscles of the body have been divided, some of the operations involving large muscular masses. Among the most important may be mentioned those applied to spinal curvature, congenital luxation of the femur, and strabismus. In one case, reported to the Académie, G. Guerin divided forty two muscles and tendons, for general deformity of the articulations, at a single sitting, no suppuration or fever resulting. Myotomy has also been applied to the *radical cure of reducible hernia*, and for *débridement* in *strangulated hernia*. In the first of these, the entire thickness of the muscles and aponeuroses constituting the canal is traversed in various directions, the resulting exudation proving an effectual obturator, as witnessed in several of the author's eleven cases. 5. *Ligaments.*—The section of these in such deformities as resist the mere division of tendons and muscles has now been performed times out of number. 6. *Vessels.* In several instances, subcutaneous incision and scarification converted vascular tumors into cicatrical tissue, and that without producing the suppuration consequent on ligatures; and various subcutaneous operations have been performed on the veins. 7. *Nerves* have been thus divided in neuralgia with advantage. 8. *Cartilages.*—M. Guerin states as a fact—of which we have met with, however, no confirmation elsewhere—that subcutaneous section of the symphysis pubis, for facilitating labor, is of common occurrence in France. 9. *Bones.*—Among these we have the subcutaneous ablation of painful exostoses, and the fracture of rickety bones in order to rectify their curvatures. M. Guerin has shown that in such curvatures the old bone is reduced to lamellæ, which are lost in bone of new formation. At the second stage of the disease, the latter is spongy and flexible, and if we make an incision half through the arch of an angular curvature, a deformity that threatened to become permanent may be at one removed.

(B.) *Punctures and Extractions.*—Under this head M. Guerin refers to three of the applications, as indicative of surgical progress: 1. The opening of large congestive abscesses.—So great is the danger attendant upon this, performed in the usual modes, that we have no example on record of success; the opening, in cases where recovery occurred, having taken place spontaneously, the pus gaining issue through a sinuous track at a distance from the seat of the collection. It is by imitating and generalizing this process of nature that the subcutaneous mode has been enabled to convert recovery into the rule and death into the exception. 2. *Thoracentesis.*—In a recent memoir, M. Guerin detailed thirty cases of this operation, deducing from them the conclusion, that the operation is devoid of all danger, and cures whenever serious complications do not exist. 3. The *extraction of foreign bodies* has been repeatedly performed with success.

(C.) Intermediate between the other two is another category of applications, comprising:—1. The *abortion of imminent phlegmon.*—M. Guerin believes that all phlegmon commences by a nucleus, a thorn as it were, in the cellular tissue, and has found, if this be divided by the subcutaneous incision, no suppuration will ensue, though the phlegmon may have reached a considerable size.

2. He has effected the *destruction of painful cervical glands* in four instances, by fixing the gland and dividing it in various directions, thus separating it from the surrounding vessels and nerves. It becomes converted into an insensible, amorphous, cicatrical tissue, which is afterwards resorbed. 3. The destruction of *painful tumours*, forming in the substance of muscles.—Under this head, too, may be noticed the section of fatty and other encysted tumours (*loupes*). Trifling as the operation is, when performed in the ordinary way, ablation has not infrequently led to fatal results. (*Gazette Médicale,*) *Med. Chir. Rev.*

